# Antimüllerian hormone levels and IVF outcomes in polycystic ovary syndrome women: a scoping review

**DOI:** 10.5935/1518-0557.20230059

**Published:** 2024

**Authors:** Luciana Carvalho Delamuta, Georges Fassolas, João Antonio Dias, Luiz Fernando de Oliveira Henrique, Felipe Passos Martins Izzo, Carlos Roberto Izzo

**Affiliations:** 1 Clínica Originare, Medicina Reprodutiva, São Paulo, Brazil

**Keywords:** polycystic ovary syndrome, antimüllerian hormone, *in vitro* fertilization, oocyte quality

## Abstract

Antimüllerian hormone (AMH) is a homodimeric glycoprotein secreted by granulosa cells from primary to large antral follicles, and it plays an important role in the regulation of early follicle growth. It is considered a reliable marker of ovarian reserve and a predictor of ovarian response to controlled stimulation. Polycystic ovary syndrome (PCOS) is an endocrine condition that affects women of reproductive age worldwide, and it is associated with high levels of AMH. PCOS patients may have worse maturation and fertilization rates compared to normo-ovulatory women. Some studies have demonstrated a positive correlation between AMH levels and qualitative aspects of assisted reproduction treatment; but it is not clear whether high levels of both serum and follicular fluid AMH in PCOS patients correlate with *in vitro* fertilization outcomes. We ran this scoping review of the literature to address this specific question. We comprehensively searched the databases PubMed and Cochrane Library until January 2023. We found that higher AMH levels are associated with higher oocyte yield, but PCOS patients tend to have fewer mature oocytes and impaired embryo quality and implantation rates. Pregnancy rates, however, are not affected by AMH levels or laboratorial outcomes. We also found that higher AMH levels are associated with worse PCOS features.

## INTRODUCTION

Antimüllerian hormone (AMH) is a homodimeric glycoprotein from the transforming growth factor-β superfamily secreted by granulosa cells from primary to large antral follicles (Desforges-Bullet *et al.*, 2010; Mashiach *et al.*, 2010; Pabuccu *et al.*, 2009). It is suggested that AMH plays an important role in the regulation of early follicle growth, with an inhibiting effect on growing follicles (Mashiach *et al.*, 2010), and suppressing the cyclical recruitment of primordial follicles (Tal *et al.*, 2014).

AMH serum levels peak around 24 years of age and decline progressively until menopause (Melado Vidales *et al.*, 2017a). It is considered a reliable marker of ovarian reserve and a predictor of ovarian response to controlled stimulation (Melado Vidales *et al.*, 2017a; Tal *et al.*, 2014). Some authors have suggested a positive correlation between AMH and oocyte quality, fertilization rates and embryo morphology (Borges *et al.*, 2017; La Marca *et al.*, 2011; Morales *et al.*, 2022).

Polycystic ovary syndrome (PCOS) is an endocrine condition that affects 5-8% of women of reproductive age worldwide (Desforges-Bullet *et al.*, 2010; Kaya *et al.*, 2010). It is the most common cause of anovulatory infertility, and it is associated with high levels of AMH, which is a consequence, not only related to the higher number of preantral and small antral follicles, but also to an increased production by individual follicles (Kaya *et al.*, 2010; Tal *et al.*, 2014).

In PCOS patients, the selection of one follicle from the increased follicular pool and its posterior dominance is impaired. The mechanisms underlying this process are still uncertain, but inhibition of the local effect of follicle-stimulating hormone (FSH) seems to play an important role. AMH inhibits the initiation of primordial follicle growth by reducing the sensitivity of follicles to FSH, thus preventing the selection of multiple follicles (Arabzadeh *et al.*, 2010). Thus, some studies have suggested that AMH plays an important role in the pathophysiology of PCOS. Serum AMH levels also appears to be related to the severity of PCOS and ovarian dysfunction, correlating with de degree of hyperandrogenism, ovulation disorder and polycystic ovary morphology (Tal *et al.*, 2014).

Although some studies have demonstrated a positive correlation between AMH levels and qualitative aspects of assisted reproduction treatment, it is known that PCOS patients may have worse maturation and fertilization rates compared to normo-ovulatory women (Fallat *et al.*, 1997; La Marca *et al.*, 2011; Mashiach *et al.*, 2010). The follicular microenvironment is also likely to be related to the success or failure of developmental competence of oocytes and embryos (Mashiach *et al.*, 2010). Fallat *et al*. (1997) demonstrated that women with PCOS had higher follicular fluid AMH levels, a higher percentage of immature oocytes, and lower fertilization rates when compared to women with endometriosis and pelvic adhesions.

It is still controversial whether high levels of both serum and follicular fluid AMH in PCOS patients can correlate well with *in vitro* fertilization outcomes such as oocyte quality, fertilization rates and pregnancy rates or if AMH levels are restricted to the prediction of ovarian reserve in these patients. Thus, the aim of the present study is to perform a literature review regarding the association between the high levels of AMH and IVF outcomes in this specific population.

## MATERIALS AND METHODS

We ran a literature review according to the PRISMA guideline (Preferred Reporting Items for Systematic Reviews and Meta-Analysis) to evaluate the study question. To identify the relevant studies, we searched the MEDLINE and Cochrane Library databases, with the last search made in January 2023 and no restriction regarding language or year of publication. Bibliographies of relevant studies identified by the search strategy and relevant reviews/meta-analyses were also searched for identification of additional studies. We retrieved the relevant articles using the search strategies described below:

MEDLINE: (((antimullerian hormone) AND (oocyte quality)) AND (embryo quality)) AND (polycystic ovary syndrome)

Cochrane Library: antimullerian hormone AND oocyte quality AND embryo quality AND polycystic ovary syndrome

The selection of manuscripts, as well as the evaluation of titles and abstracts, were conducted by two blinded researchers (L.C.D. and C.R.I) working independently. After this initial screening, the remaining articles were further evaluated to be included or not in the review.

## RESULTS

**Figure 1:**
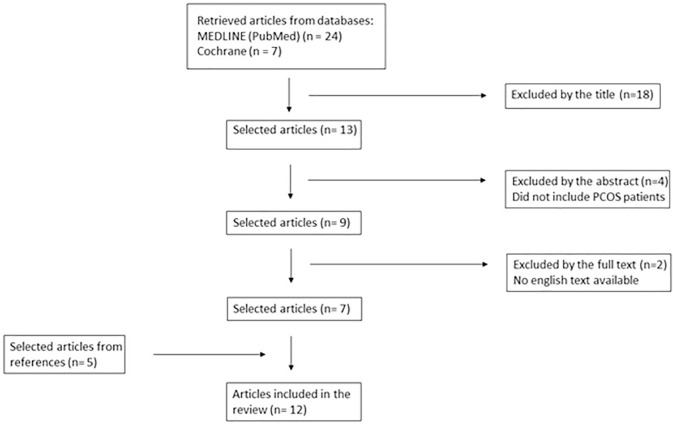
Flowchart of the studies included.

The search process to identify and select the studies is presented in Figure 1. We found a total of 31 studies, of which 12 studies were included in the final review. Eighteen studies were excluded after an initial screening based on the titles. Four other studies were excluded after abstract reading for not individualizing patients with PCOS. Two other studies were excluded after full text reading because they were written in Chinese and no English text version could be retrieved. Five more studies were included after we searched the References of the studies already included. Table 1 summarizes the studies included in the review.

**Table 1:** Summary of the studies included.

Study	Study type	Objective	Population	Number of individuals	Main results
Fallat *et al*., 1997		To determine Mullerian inhibiting substance (MIS) levels in follicular fluid (FF) and sera of IVF patients.	Infertile patients undergoing IVF treatment with tubal factor (20), PCOS (17) and endometriosis (29)	66	Levels of AMH in FF and sera of PCOS patients were significantly higher than those in the other patients. Also, the percentage of immature oocytes retrieved in PCOS were significant higher and the fertilization rates were significantly lower than in the other groups.
Desforges-Bullet *et al*., 2010	Prospective	To confirm the increased levels of AMH in preovulatory follicles from PCOS patients and to study the role of other hormones involved in folliculogenesis in this increased secretion.	22 PCOS patients and 20 controls undergoing IVF treatment.	42	AMH levels were significantly increased and FSH levels significantly decreased in FF from PCOS patients. Mean androstenedione, hCG, E2 and progesterone did not differ.
Mashiach *et al*., 2010	Prospective	To test whether the FF concentration of AMH is associated with oocyte maturation, fertilization rate, and embryonic development in patients with PCOS undergoing IVF.	11 PCOS patients and 12 controls.	23	Mean FF AMH level was smaller in PCOS patients compared with controls. In PCOS patients the mean AMH level of good quality embryos was smaller than that of poor quality embryos. No significant correlation was observed between FF AMH levels and oocyte maturation, fertilization, or cleavage rate.
Pabuccu *et al*., 2009	Prospective	To investigate the relationship of serum and follicular-fluid AMH concentrations on the day of oocyte retrieval and reproductive outcome in PCOS patients undergoing assisted reproduction.	Infertile PCOS patients	80	Clinical pregnancy rates, embryo implantation rates and fertilization rates were markedly different among the low, moderate and high follicular-fluid AMH groups but not among the different serum AMH concentration groups.
Arabzadeh *et al*., 2010	Prospective	To compare the relationship between serum or intrafollicular AMH levels and IVF outcomes in women with and without PCOS.	26 women with PCOS and 42 normo-ovulatory controls	68	Median serum basal AMH and FF AMH levels were significantly higher in the PCOS group as compared to controls. In both groups, serum basal AMH levels showed a positive correlation with number of oocytes retrieved. In the control group, there was a positive relationship between serum basal AMH levels and percentage of matured oocytes and implantation rate.
Kaya *et al*., 2010	Prospective clinical trial	To determine the possible relationship between serum AMH concentrations on day 3 and controlled ovarian stimulation and reproductive outcomes in women with PCOS.	Patients with PCOS and infertility	60	The CPR, IR and FR increased progressively with the increase in day-3 AMH serum concentrations.
Xi *et al.*, 2012	Prospective cohort	To investigate if day 3 serum AMH concentrations day 3 could predict controlled ovarian stimulation and reproductive outcomes in women with PCOS.	PCOS patients undergoing their first IVF cycle	164	E2 levels on trigger day and oocytes retrieved significantly increased with increasing AMH levels. Implantation rate and clinical pregnancy rate were lower in the higher AMH group. FR and number of good quality embryos were comparable between groups.
Yilmaz *et al*., 2012	Prospective sequential cross sectional study	To investigate the follicular fluid concentrations of AMH and its effect on assisted reproductive technology outcome in non-obese, non-hyperandrogenomic PCOS patients	Primary infertile patients with PCOS (16), male factor (19) and unexplained infertility (19)	54	FF AMH levels were higher in patients with PCOS but did not reach statistical significance. FF AMH was positively correlated with oocyte, 2PN and embryo number. FF AMH levels weren’t different between the groups who were pregnant or not.
Tal *et al.*, 2014	Retrospective cohort study	Characterize a population of women with elevated AMH (>5ng/mL).	Women with serum AMH > 5ng/mL	134	Greater than 97% of women with ultrahigh AMH (>10 ng/mL) had PCOS and also greater prevalence of PCOM and oligomenorrhea. Serum AMH correlated positively with LH, total testosterone, DHEAS and showed strong predictive ability for the presence of amenorrhea. Women with AMH >10 ng/mL showed higher rates of ovarian hyperstimulation syndrome and clinical pregnancy rates compared with women with AMH 5-10 ng/mL.
Chen *et al.*, 2017	Prospective study	Evaluate associations between basal serum and FF AMH levels and IVF outcomes in PCOS patients.	Infertile patients with PCOS (59), tubal and male factor (120)	179	Median serum and FF AMH level was higher in PCOS patients than in controls. Oocyte maturation and high-quality embryo rates were lower in PCOS patients than in controls, but both groups had similar fertilization, implantation, clinical pregnancy, and newborn rates. Peak E2 and FF AMH levels were independent predictors of oocyte number.
Melado Vidales *et al*., 2017b	Prospective observational cohort study	To assess the correlation between COH outcome parameters and AMH serum levels during IVF treatment in women with varying ovarian reserve levels.	Infertile patients undergoing IVF with low (11)/normal (16) ovarian reserve and PCOS (19).	46	AMH was significant correlated with AFC, number of follicles >11mm on hCG trigger day, number of oocytes retrieved and MII oocytes. AMH on hCG trigger day was correlated with top quality embryos.

Most of the studies were prospective, while only one was a retrospective cohort study. Eight of the twelve studies reported the follicular fluid levels of AMH (FF AMH). Seven of these eight studies compared PCOS patients with control normo-ovulatory patients. Five of them reported higher FF AMH in PCOS patients, while one study found lower FF AMH in PCOS patients, and another study did not find differences in FF AMH (Arabzadeh *et al.*, 2010; Chen *et al*., 2017; Fallat *et al.*, 1997; Mashiach *et al.*, 2010; Melado Vidales *et al.*, 2017b; Pabuccu *et al.*, 2009; Yilmaz *et al.*, 2012).

Seven studies reported the number of oocytes retrieved (Arabzadeh *et al.*, 2010; Chen *et al.*, 2017; Fallat *et al.*, 1997; Mashiach *et al.*, 2010; Melado Vidales *et al.*, 2017a; Yilmaz *et al.*, 2012, Xi *et al.*, 2012). Fallat *et al.* (1997) found a higher number of immature oocytes in PCOS patients compared to patients with tubal factor infertility and endometriosis. Overall, there is a positive correlation between AMH levels and the number of oocytes retrieved in all patients, but not necessarily for the number of mature oocytes (Fallat *et al.*, 1997). Mashiach *et al*. (2010) found no correlation between FF AMH and oocyte maturation in PCOS patients compared to controls. Arabzadeh *et al.* (2010) showed that higher AMH levels were associated with higher oocyte yield, but a positive relationship between serum AMH levels and mature oocytes was observed only in the non-PCOS group. Chen *et al.* also reported a lower oocyte maturation rate in PCOS patients (Chen *et al.*, 2017).

Eight studies analyzed fertilization rates (FR), five of them comparing PCOS patients to normo-ovulatory patients, while the remaining three studies addressed PCOS patients only (Chen *et al.*, 2017; Fallat *et al.*, 1997; Kaya *et al.*, 2010; Mashiach *et al.*, 2010; Melado Vidales *et al.*, 2017b; Pabuccu *et al.*, 2009; Yilmaz *et al.*, 2012; Xi *et al.*, 2012). Fallat *et al.* (1997) were the only authors that found significantly lower fertilization rates in PCOS patients compared to controls. Pabuccu *et al.* (2009) showed that in PCOS patients the FR increased with higher FF AMH levels but did not correlate with serum AMH levels. Kaya *et al.* (2010) on the other hand, demonstrated a progressive increase in FR with higher serum AMH concentrations.

Five studies addressed the embryo quality and only one of them found PCOS patients had lower rates of high-quality embryos (Chen *et al.*, 2017; Mashiach *et al.*, 2010; Melado Vidales *et al.*, 2017a; 2017b; Xi *et al.*, 2012). Melado Vidales *et al.* (2017a) demonstrated serum AMH levels on hCG trigger day were correlated with the number of top-quality embryos obtained in all the patients studied. The same authors, however, found no significant between-group differences for AMH levels in FF nor for FR, number of TQE or implantation rates when comparing PCOS patients to patients with low/normal ovarian reserve (Melado Vidales *et al.*, 2017b).

Six studies reported implantation rates (IR) and the results were discrepant (Arabzadeh *et al.*, 2010; Chen *et al.*, 2017; Kaya *et al.*, 2010; Melado Vidales *et al.*, 2017b; Pabuccu *et al.*, 2009; Xi *et al.*, 2012). The two studies that compared PCOS patients with normo-ovulatory controls did not find significant differences in IR between groups (Chen *et al.*, 2017; Melado Vidales *et al.*, 2017b). Of the studies that addressed only PCOS patients, Pabuccu *et al*. (2009) found IR were markedly increased with higher follicular-fluid AMH but not among the different serum AMH concentration groups. On the other hand, Kaya *et al.* (2010) showed IR increased progressively with the increase in day-3 AMH serum concentrations while Xi *et al*. (2012) found lower IR with higher serum AMH levels.

Clinical pregnancy rates (CPR) were reported in five of the twelve studies (Chen *et al.*, 2017; Kaya *et al.*, 2010; Pabuccu *et al.*, 2009; Tal *et al.*, 2014; Xi *et al.*, 2012). Only one of them compared PCOS patients with normo-ovulatory controls and found similar rates, although the FF AMH levels were markedly higher in PCOS patients (Chen *et al.*, 2017). Xi *et al.* (2012) and Kaya *et al.* (2010) studied only PCOS patients and showed conflicting data. While the first group of authors found CPR were lower in patients with higher serum AMH levels, the former demonstrated the opposite. Pabuccu *et al.* (2009) on the other hand, showed a positive correlation between CPR and FF AMH, but not serum AMH concentrations. A retrospective cohort study evaluated patients with serum AMH levels higher than 10 ng/mL and demonstrated a 97% prevalence of PCOS in this population. Moreover, these patients had a significantly higher CPR compared with patients who have AMH levels between 5 and 10 ng/mL (Tal *et al.*, 2014).

## DISCUSSION

The present study consisted of a scoping review that evaluated the association between AMH levels and IVF outcomes in women with infertility and PCOS. Our study showed that AMH levels in both serum and FF are higher in PCOS patients compared to women with other infertility factors. Regarding the laboratorial and pregnancy outcomes in IVF treatment, the studies analyzed were unanimous in concluding that PCOS women have a higher oocyte yield; however, whether this translates into better maturation, fertilization, implantation, clinical pregnancies, and live birth rates is still controversial.

AMH is a well-known marker of ovarian reserve and has been proven to be positively associated with ovarian response in controlled stimulation and, therefore, oocyte yield (Brodin *et al.*, 2013). In PCOS the serum AMH levels are up to 5-fold higher than in normo-ovulatory women (Xi *et al.*, 2012), mainly due to an increased in AMH production by each individual follicle rather than a higher number of follicles (Fanchin *et al.*, 2005). In the past years, some studies have analyzed the AMH levels in follicular fluid during oocyte pickup (OPU) after controlled ovarian stimulation. As expected, most of them showed higher levels in PCOS patients (Arabzadeh *et al.*, 2010; Chen *et al.*, 2017; Fallat *et al.*, 1997; Pabuccu *et al.*, 2009; Yilmaz *et al.*, 2012). Mashiach *et al.* (2010) found lower mean levels of AMH in FF of PCOS patients compared to normo-ovulatory controls; and Melado Vidales *et al.* (2017b) found no between-group differences when comparing PCOS women to patients with low and normal ovarian reserve. It is worth noting, though, that both studies are limited by small sample sizes. The AMH production by granulosa cells decreases during oocyte maturation, and the development of small antral follicles into mature follicles during controlled ovarian stimulation is associated with a dramatic decrease in serum AMH levels. Fanchin *et al.* (2005) demonstrated that FF AMH levels were 3 times higher in follicles 8-12mm than 16-20mm and these levels were positively associated with AFC and oocyte yield.

PCOS patients have a higher number of oocytes collected after OPU, but this does not necessarily translate into a higher number of mature oocytes. Fallat *et al.* (1997) demonstrated that immature oocytes rate was higher in PCOS patients and Arabzadeh *et al*. (2010) found that, although the serum and FF AMH levels were higher in PCOS compared to normo-ovulatory controls, the positive correlation between AMH concentrations and mature oocytes was fund only in the control group. A prospective study by Ebner *et al.* (2006) divided patients according to their AMH serum levels and found that serum AMH <1.66ng/mL and >4.52ng/mL were associated with oocytes of lower quality. The study included patients undergoing ICSI, most of them for male factor infertility and did not address PCOS patients specifically. However, AMH levels higher than 5ng/mL are commonly associated with PCOS, so it is possible that part of the cohort of patients had the syndrome.

PCOS is characterized by an abnormal folliculogenesis and failure in selecting a dominant follicle, which is reflected by high levels of AMH (Desforges-Bullet *et al.*, 2010). It is known that AMH has an inhibitory effect on FSH-induced aromatase expression in granulosa cells (Tal *et al.*, 2014; Xi *et al.*, 2012). This reduces the follicle sensibility to FSH, which could lead to impaired oocyte maturation during controlled ovarian stimulation. Indeed, Desforges-Bullet *et al.* (2010) found lower FF FSH levels in PCOS patients compared to controls and, although the clinical pregnancy rates were similar between the groups, FF AMH was lower in patients who were pregnant. It is not clear whether the deleterious effect AMH has on FSH function could impact on fertilization rates. Chen *et al.* (2017) observed higher AMH levels on both serum and follicular fluid of PCOS patients compared to male and tubal factor infertility, and found no differences in fertilization rates. However, the oocyte maturation and top-quality embryo rates were significantly lower in PCOS patients (Chen *et al.*, 2017). Fallat *et al.* (1997) showed the percentage of immature oocytes retrieved in PCOS were significantly higher, and the fertilization rates were significantly lower compared to patients with tubal factor infertility and endometriosis. On the other hand, Kaya *et al.* (2010) analyzed only PCOS patients and demonstrated a positive correlation between AMH levels and the number of mature oocytes, fertilization, implantation, and clinical pregnancy rates. All studies are limited by a small sample size and do not consider the different PCOS phenotypes.

One of the many factors limiting the clinical pregnancy rates after assisted reproductive treatments is the embryo quality. The disruption in folliculogenesis observed in PCOS patients leads to lower fertilization and cleavage rates in this population, and could potentially impair embryo quality and, thus, pregnancy rates (Mashiach *et al.*, 2010). While Mashiach *et al.* (2010) found that in PCOS patients the FF AMH was related to lower top-quality embryos compared to controls. Xi *et al.* (2012) did not report differences on embryo quality when separating PCOS patients by the serum AMH levels. Though the studies evaluated AMH concentrations in different fluids, it is already known that both are elevated in women with PCOS compared to normo-ovulatory women (Mashiach *et al.*, 2010; Pabuccu *et al.*, 2009; Xi *et al.*, 2012). Chen *et al*. (2017) reported a smaller number of top-quality embryos in PCOS patients when compared to patients with tubal and male factor infertility, and higher levels of FF AMH. Despite the lower number of top-quality embryos in PCOS patients, the implantation and pregnancy rates were similar in all groups. Notwithstanding, the authors did not disclose the number of embryos available for transfer in each group. It is possible that PCOS patients have a higher number of embryos, and this could overcome the embryo quality problem. Moreover, all control patients received a fresh embryo transfer, while some of the PCOS patients had a frozen embryo transfer due to the high risk of ovarian hyperstimulation syndrome. Ciepiela *et al.* (2019) analyzed data from women with several infertility factors undergoing ICSI treatment, and found that FF AMH was higher, and FF FSH was lower in follicles in which the oocyte developed into a top-embryo quality. Whether this association remains true for exclusively PCOS patients remains elusive. As already demonstrated by Melado Vidales *et al.* (2017a; 2017b), serum AMH on hCG trigger day in PCOS patients was associated to embryo quality and not FF AMH. Notwithstanding, the studies are limited by their small sample sizes (Melado Vidales *et al.*, 2017a; 2017b).

CPR and live birth rates are the most valuable outcomes for patients undergoing assisted reproductive treatment. La Marca *et al.* (2011) have already assessed the use of AMH in a model to predict live birth rates in women undergoing IVF treatment. They found statistically significant lower odds of live birth for increasing age and decreasing AMH (La Marca *et al.*, 2011). The study participants were included regardless of infertility factor, and there was no subgroup analysis for PCOS women. Tal *et al.* (2014) analyzed infertile patients with serum AMH levels higher than 5ng/mL and found that ultrahigh levels (> 10ng/mL) could predict PCOS with 97% accuracy. Women with AMH >10 ng/mL had higher rates of ovarian hyperstimulation syndrome and CPR compared with women with AMH 5-10 ng/mL. Also, women with higher AMH levels have more frequency of oligomenorrhea and polycystic ovary morphology. Serum AMH correlated positively with LH, total testosterone, DHEAS levels and showed strong predictive ability for the presence of amenorrhea (Tal *et al.*, 2014). It is possible that the AMH concentrations reflect the severity of PCOS. As already demonstrated, higher levels in serum and follicular fluid are associated with lower oocyte maturity rates in some studies (Arabzadeh *et al.*, 2010; Ebner *et al.*, 2006; Fallat *et al.*, 1997). This maturation impairment is not necessarily associated with lower clinical pregnancy rates, as demonstrated by Pabbucu *et al.* (2009) and Kaya *et al.* (2010) showing higher pregnancy rates as FF AMH and serum AMH increased, respectively. On the other hand, Xi *et al*. (2012) reported lower CPR in PCOS patients with serum AMH levels >8.82ng/mL. This difference, however, was only close to statistical significance and the study sample size is small to draw any definitive conclusion (Xi *et al.*, 2012). Chen *et al.* (2017) also found no differences in CPR when comparing PCOS patients with tubal and male factor infertility. The numbers of mature oocytes and top-quality embryos were, however, lower in PCOS patients, with a median serum and FF AMH higher.

All studies that compared PCOS patients to normo-ovulatory controls unanimously concluded that oocyte retrieval is higher in the first group. Although the oocyte maturation and fertilization rates can differ between patients, leading to lower top-quality embryos, CPR were not affected by laboratorial outcomes. One possible explanation for the impaired oocyte maturation and embryo development is that the higher AMH levels reflect more severe PCOS characteristics, such as worse metabolic features and hyperandrogenism. Patients with higher AMH levels may also have higher LH levels, which can result in higher intrafollicular hyperandrogenism, leading to impaired oocyte development and, therefore, affect embryo quality. The inhibitory effect AMH has on FSH-induced aromatase expression in granulosa cells may also be responsible for lower follicular sensibility to FSH and worse ovarian stimulation outcomes.

It is widely known that in assisted reproduction, the more oocytes a patient has, the better odds of getting a pregnancy. The higher levels and both serum and follicular fluid AMH levels can have a deleterious impact on laboratorial features, but it is possible that the higher oocyte numbers are able to overcome these disadvantages. Therefore, although AMH concentrations can predict worse features of PCOS, it can also be a marker of higher oocyte yield. Whether the different phenotypes and severity of PCOS could have a negative impact on pregnancy outcomes after assisted reproduction is a matter for further studies.

## CONCLUSION

This review summarized the available evidence on AMH levels and reproductive outcomes in PCOS infertile women. We concluded that women with PCOS have higher serum and follicular fluid AMH levels, and it is a predictor of higher oocyte yield. However, oocyte and embryo quality are usually worse in these patients. The pregnancy rates are not affected by laboratorial outcomes, which shows that higher oocyte number can overcome the embryo quality problem. Moreover, AMH levels concentration can predict worse features of PCOS.
